# Observations on the Convulsion Incidental to Seamen from Excessive Drinking

**Published:** 1830-05

**Authors:** James Wallaw

**Affiliations:** Assistant Surgeon R.N.


### CONVULSION.

*Observations on the Convulsion incidental to Seamen from excessive Drinking*. By JAMES WALLAW, Assistant Surgeon R.N.

IT is well known that one of the most frequent exciting causes of spasmodic or convulsive action, is the existence of some irritation in the primæ viæ. To give effect, however, to this exciting cause, there must, in all probability, be a predisposition to the disease in the habit of the individual. Thus, we observe that convulsive diseases are much more common in infants and children than in those further advanced in life; again, they are more common in the female than in the male; and, lastly, there are certain male constitutions infinitely more liable to them than others. Thus it is evident that a certain peculiarity of system is requisite, a certain irritability of fibre, or whatever else we may please to call it, must be in existence, to give the patient the convulsive liability; and then, either by the presence of irritating food in the stomach, or the accumulation of feculent matter in the bowels, or a vitiated state of the secretions, or any other morbid or inordinate stimulus to the first passages, the disease is instantly called into action.

Now, there prevails amongst seamen, too generally, a fondness for inebriating liquors, and it is customary with many of them, as often as an opportunity offers, to indulge to excess. This may be attributed, in some measure, to the privations which they are obliged to suffer: they are frequently for a long period, at sea, kept almost without enjoyment; and it is natural to suppose that, when they return to port, with more money in their possession than they can well dispose of, they will be led into temptation. But, whatever may be the cause or causes of the failing, it is certain that it exists, and, as a consequence, it brings upon them much misery, both in a pecuniary point of view and as regards their bodily health. They bring upon themselves, by their excesses at home and abroad, many severe maladies; and one of these is convulsion.

During a period of three years, on a station where inebriating liquors were cheap, and consequently within reach of the seamen, I had ample opportunities of witnessing the deleterious effects of drinking to excess. It was customary in that station to allow, on a particular day of the week, a certain portion of the seamen to visit their friends and acquaintances in the neighbouring ships, and as certainly as they went on that visit, so certainly did the majority of them return to their ships in a state of intoxication; some of them in a complete state of insensibility. Many, no doubt, pursued this course, to all appearance, with perfect impunity; but serious disease was the consequence to others, and to some violent convulsion or spasm was almost a sure consequence.

The disease, proceeding from this cause, in general makes its attack very suddenly. The person is, perhaps, observed to be in a state of intoxication, but no one thinks of paying him any attention, as his appearance indicates at first nothing above common drunkenness. By and by he becomes restless; if he was asleep, he becomes watchful; the countenance puts on a disturbed and anxious appearance; he wishes to shift continually from his position; and suddenly the convulsion ensues, and proceeds with such violence as to demand the help of two or three strong persons to keep the patient secure. The spasmodic action seems to exist primarily and most severely in the abdominal and thoracic muscles, extending from thence to the muscles of the neck, lower jaw, &c.; but, in fact, the whole muscular apparatus is in one violent convulsion. The limbs are agitated in every direction; attempts are made to raise the head forcibly from the ground, and as violently to strike it back again; the teeth are gnashed together; frothy saliva flows from the mouth; the eyelids are either shut, and the pupil contracted, while the patient mutters incoherently, or they are widely opened, the pupil dilated, and the countenance has altogether a furious aspect. This excessive degree of spasm continues for probably a minute or more, when it subsides, and the patient is for a few minutes quiescent. During this period he is nearly altogether insensible, although, by rousing him, he may be made to answer questions indistinctly. Previous to the return of spasm, he again becomes restless; he then either mutters to himself, or screams aloud, or laughs sardonically; and thus does he continue, alternately quiescent and convulsed, for a period of from one to three hours (varying according to circumstances) until the system, as if fairly worn out in the struggle, becomes permanently quiescent, and sleep overtakes the unfortunate sufferer. In almost every case the pulse is quickened, and considerably fuller than natural. It rarely happens that any attempt is made to protrude the tongue during the spasm.

So far as I have observed, there is but little danger to be apprehended, either during the attack or after it. On the following day the patient, although somewhat languid and depressed, is generally able to return to his duty, and in a day or two more he is as well as if nothing had happened to him. Neither can I say, except in one case, that I have seen a repetition of the attack, except from a repetition of the exciting cause. In that particular instance, the patient had three or four attacks in the course of as many days, but none of them were to compare in violence with the first. But though the disease is not dangerous, yet it can scarcely be doubted that, by being frequently repeated, even a robust constitution would ultimately be seriously injured.

That the disease in question is produced by the inordinate quantity of stimulating fluids taken into the stomach, is evident from the circumstance that, on the station already alluded to, it was seldom met with except on the night on which the men had an opportunity of indulging. If we had an occasional case at other times, it could generally be traced to the same origin. At the same time I have reason to suppose that any irritation of mind, such as grief or anxiety, tends considerably to the production of the ailment; and I am also satisfied that indulging plentifully in bad wine has an infinitely more pernicious effect than the same indulgence in spirituous liquors. It was chiefly wine which the men, at the period I speak of, could obtain, which wine was always of an inferior quality, frequently much acidified; in fact, almost in a state of vinous fermentation; consequently, its effects on the system generally must have been exceedingly deleterious. I therefore attribute, in a great measure, to this adulterated and decomposed wine, the prevalence of convulsion at that period; for I have never seen, either before or since, even when drunkenness has prevailed to a considerable extent, if that drunkenness was the effect of tolerably good spirits, spasmodic cases at all in the same proportion.

To prove the liability which exists in certain constitutions to the complaint, I may mention that there were a few individuals who were subjected to an attack every time they indulged: as certainly as they became intoxicated, so certainly did they suffer; others, again, had one or two attacks, and escaped ever after; others, of course, (and the majority,) escaped altogether.

The individuals evidently most disposed to the disease were the young and the robust. Between the ages of twenty and thirty-five, there were more attacked by it than either above or under that period. Those, however, who had by no means vigorous constitutions were frequently its subjects.

The treatment of the complaint is simple, and must at once be evident from the explanation already given of its exciting cause. Seeing that the convulsion is positively induced by the over-distended and stimulated state of the stomach, our first effort must be towards the unloading of that viscus. This is best done by administering half a drachm of the sulphate of zinc, or four grains of tartrate of antimony, in an ounce or two of water. After waiting fifteen or twenty minutes, if vomiting be not induced, (and be it remembered, that in these cases there is a remarkable torpidity of stomach,) we may repeat the dose, and having waited twenty minutes longer without effect, if the patient can at all bear it, the sooner we detract blood the better. Take thirty ounces of blood rapidly from the arm, and probably before the bleeding is finished, at all events very soon after, vomiting takes place. When this happens, the spasm very soon gives way. As soon as the nausea begins to appear, the attacks of spasm become both less frequent and severe, and by the time the stomach is fairly emptied, the patient is generally free from the ailment, and soon after falls asleep. It might be advisable to substitute for a part of the antimony a portion of ipecacuan, but, from its nauseous taste, the patient often refuses to swallow it: he spits it out even after it is put into his mouth; and, indeed, it is sometimes so difficult to get him to swallow any thing, that we are obliged to give up the attempt, and have recourse to venesection alone. Besides these means, we might, in particular cases, have recourse to the warm bath, cold affusion, tobacco injection, &c. When much depression follows the attack, camphor mixture, with carbonate of ammonia, will be the most serviceable medicines.

I have sometimes thought that, by the timely application of the stomach-pump, we might in many cases prevent the attack of convulsion altogether. When we are told that a person whom we knew to be predisposed to the ailment is in a state of inebriation, would it not be advantageous to rid the stomach at once of its deleterious contents by means of this instrument? It would be nearly impossible to put the pump in use after the disease had shown itself, but previous to its accession it might be done with perfect ease. I have even thought that in all cases of excessive drunkenness, whether there was a liability to any ailment or not, this excellent instrument might be used with propriety. Might it not, therefore, be an advantageous appendage to the naval surgeon's instruments?

Before concluding, I have to observe, that at all times we ought to be cautious as to the vessel out of which we give the patient drink or medicine in this disease. Of course, we always seize the period of quiescence to offer him whatever he may have to swallow, but the convulsion frequently attacks at the moment he is drinking, and, if we are not very much on the alert indeed, the vessel is violently grasped between the teeth. I once saw a considerable portion of a glass tumbler in this way taken into the mouth, and afterwards chewed and swallowed. Fortunately in this case no serious injury was done; the lips and gums were merely slightly lacerated; but the consequences might have been worse. The best way is to give the medicine either from a horn or metal vessel, or give it by spoonsful, without introducing the spoon between the teeth.

*H. M. Ship Gloucester.*

---

DULCAMARA.

